# Caterpillar–parasitoid interactions: species-specific influences on host microbiome composition

**DOI:** 10.1093/femsec/fiae115

**Published:** 2024-08-20

**Authors:** Gabriele Gloder, Mitchel E Bourne, Maximilien A C Cuny, Christel Verreth, Sam Crauwels, Marcel Dicke, Erik H Poelman, Hans Jacquemyn, Bart Lievens

**Affiliations:** CMPG Laboratory for Process Microbial Ecology and Bioinspirational Management (PME&BIM), Department M2S, KU Leuven, Willem De Croylaan 46, B-3001 Leuven, Belgium; Leuven Plant Institute (LPI), KU Leuven, Kasteelpark Arenberg 31, B-3001 Leuven, Belgium; Laboratory of Entomology, Wageningen University, Droevendaalsesteeg 1, 6708 PB Wageningen, The Netherlands; Laboratory of Entomology, Wageningen University, Droevendaalsesteeg 1, 6708 PB Wageningen, The Netherlands; CMPG Laboratory for Process Microbial Ecology and Bioinspirational Management (PME&BIM), Department M2S, KU Leuven, Willem De Croylaan 46, B-3001 Leuven, Belgium; Leuven Plant Institute (LPI), KU Leuven, Kasteelpark Arenberg 31, B-3001 Leuven, Belgium; CMPG Laboratory for Process Microbial Ecology and Bioinspirational Management (PME&BIM), Department M2S, KU Leuven, Willem De Croylaan 46, B-3001 Leuven, Belgium; Leuven Plant Institute (LPI), KU Leuven, Kasteelpark Arenberg 31, B-3001 Leuven, Belgium; Laboratory of Entomology, Wageningen University, Droevendaalsesteeg 1, 6708 PB Wageningen, The Netherlands; Laboratory of Entomology, Wageningen University, Droevendaalsesteeg 1, 6708 PB Wageningen, The Netherlands; Leuven Plant Institute (LPI), KU Leuven, Kasteelpark Arenberg 31, B-3001 Leuven, Belgium; Laboratory of Plant Conservation and Population Biology, Biology Department, KU Leuven, Kasteelpark Arenberg 31, B-3001 Leuven, Belgium; CMPG Laboratory for Process Microbial Ecology and Bioinspirational Management (PME&BIM), Department M2S, KU Leuven, Willem De Croylaan 46, B-3001 Leuven, Belgium; Leuven Plant Institute (LPI), KU Leuven, Kasteelpark Arenberg 31, B-3001 Leuven, Belgium

**Keywords:** *Cotesia glomerata*, *Cotesia rubecula*, microbiome, parasitic wasp, parasitism, *Pieris brassicae*, *Pieris rapae*

## Abstract

There is increasing evidence that host–parasitoid interactions can have a pronounced impact on the microbiome of host insects, but it is unclear to what extent this is caused by the host and/or parasitoid. Here, we compared the internal and external microbiome of caterpillars of *Pieris brassicae* and *Pieris rapae* parasitized by *Cotesia glomerata* or *Cotesia rubecula* with nonparasitized caterpillars. Additionally, we investigated the internal and external microbiome of the parasitoid larvae. Both internal and external bacterial densities were significantly higher for *P. brassicae* than *P. rapae*, while no differences were found between parasitized and nonparasitized caterpillars. In contrast, parasitism significantly affected the composition of the internal and external microbiome of the caterpillars and the parasitoid larvae, but the effects were dependent on the host and parasitoid species. Irrespective of host species, a *Wolbachia* species was exclusively found inside caterpillars parasitized by *C. glomerata*, as well as in the corresponding developing parasitoid larvae. Similarly, a *Nosema* species was abundantly present inside parasitized caterpillars and the parasitoid larvae, but this was independent of the host and the parasitoid species. We conclude that parasitism has pronounced effects on host microbiomes, but the effects depend on both the host and parasitoid species.

## Teaser

Parasitic wasps alter the microbiome of their host, but effects depend both on the host and wasp species.

## Introduction

Most insects harbour a variety of microorganisms whose diversity and roles are only recently being better understood (Engel and Moran 2013, Douglas 2015, Muñoz-Benavent et al. 2021). Their internal microbiomes either comprise a stable assemblage of microorganisms that can be consistently detected in larval and adult hosts (Bright and Bulgheresi 2010, Engel and Moran 2013) or harbour transient gut microbes (Hammer et al. 2017, 2019). These microorganisms may play important roles in insect behaviour, food digestion, nutrition, detoxification, and protection of their host against abiotic stress, pathogens, and parasites (Douglas 2015). Similarly, the external surfaces of insects (i.e. the exoskeleton) are commonly inhabited by microorganisms. Unlike the internal microbiome, the external insect microbiome is often composed of a diverse group of nonspecialized environmental microorganisms that vary significantly with geographic location and habitat (Park et al. 2019).

The composition and diversity of insect microbiomes are affected by a wide range of factors, including host phylogeny, life stage, diet, and habitat (Behar et al. 2008, Ottesen and Leadbetter 2010, Yun et al. 2014, Chen et al. 2016, Shao et al. 2024). Additionally, there is increasing evidence that the microbial community composition and diversity in insects is strongly influenced by host–parasite interactions (but see Liu et al. 2020). Parasites like helminths and protozoa residing in the insect gut may alter the composition of the gut microbiome (Fredensborg et al. 2020). Similarly, insect–parasitic nematodes (Vicente et al. 2016) and koinobiont parasitic wasps (parasitoids) have been shown to modify the internal microbiome of their hosts (Polenogova et al. 2019, Cavichiolli de Oliveira and Consoli 2020, Gao et al. 2021, Gloder et al. 2021, Zhang et al. 2022, Wang et al. 2023, Gwokyalya et al. 2024).

Koinobiont parasitoids are important secondary consumers in arthropod communities and key natural enemies of agricultural pests. They deposit their eggs inside or outside their hosts, and their larvae parasitize the hosts while keeping them alive for a certain period of time (Schafellner et al. 2004). The parasitoid larvae can alter host behaviour such as food preference (Smilanich et al. 2011) and food intake and utilization (Rossi et al. 2014), which in turn may impact the diversity and composition of the host insect microbiomes (Yun et al. 2014). Furthermore, adult parasitoids may transfer some of their microbiota during oviposition and alter the microbiome of their host both directly and indirectly (Douglas 2015, Gloder et al. 2021, Gwokyalya et al. 2024). Research has demonstrated that parasitoid symbionts and venom injected with the wasp eggs can manipulate host physiology and suppress the host immune system to benefit the survival of the parasitoid’s offspring (Strand and Pech 1995). At the same time, this process may also affect the regulation of gut microbes, thereby indirectly changing the host microbiome (Cavicchiolli de Oliveira and Consoli 2020).

Therefore, we hypothesize that the internal microbiomes of parasitized insects are to a large extent determined by characteristics of both the host and parasitoid species. Similarly, we predict that the microbiome of the parasitoid larvae that develop in the host is determined by features of both the host and parasitoid species. Conversely, given that parasitoids parasitize the interior of their hosts, we expect that they do not, or to a lesser extent, affect the external microbiome of their host (Gloder et al. 2021, Bourne et al. 2023). To test these hypotheses, we compared both the internal and external microbiomes of parasitized and nonparasitized hosts and examined whether differences were mainly driven by the host, the parasitoid, or a combination of both. Furthermore, we asked which microbes were commonly transferred to the hosts through parasitism. We also assessed the microbiomes of the developing parasitoid larvae and investigated to which extent they are influenced by the host, the parasitoid or their interaction. To this end, we used the large cabbage white *Pieris brassicae* and the small cabbage white *Pieris rapae* (Lepidoptera: Pieridae) and their main koinobiont endoparasitoids *Cotesia glomerata* and *Cotesia rubecula* (Hymenoptera: Braconidae) as study species. Previous research using *P. brassicae* and *C. glomerata* has shown that parasitism by *C. glomerata* has a major impact on the host microbiome (Gloder et al. 2021, Bourne et al. 2023). However, the specific contributions of the host and parasitoid species to these alterations remain to be fully elucidated.

## Materials and methods

### Study species

*Pieris rapae* has a natural range across Europe, North Africa, and Asia, but has also been found in North America, Australia, and New Zealand. In contrast, *P. brassicae* is less widely distributed and mainly occurs in Europe, Asia, and North Africa. Both species are important pests on many crop species belonging to the family Brassicaceae such as cabbage, cauliflower, Brussels sprouts, and rape. *Pieris brassicae* lays eggs in clusters of 10–100 eggs whereas *P. rapae* lays single eggs, leading to gregarious and solitary larvae, respectively (Davies and Gilbert 1985). *Cotesia glomerata* is a gregarious koinobiont wasp that parasitizes a wide range of caterpillars of pierid butterflies, but *P. brassicae* and *P. rapae* are its main hosts (Brodeur et al. 1996). On average, adult females of *C. glomerata* lay around 20 eggs in a host caterpillar per oviposition event (Brodeur et al. 1996). In contrast, *C. rubecula* is a solitary parasitoid and has long been considered to be specific to *P. rapae* (Shenefelt 1972), but it may also parasitize *P. brassicae* larvae (Brodeur et al. 1996, 1998). Once the egg(s) hatch, the larvae of both parasitoid species feed on the caterpillar’s haemolymph while the caterpillars are still alive. Larvae of *C. glomerata* emerge from their caterpillar host ~15–20 days after parasitization, while it takes around 10–15 days for *C. rubecula* larvae to emerge and pupate outside of the host. At that time caterpillars are generally in the last instar (L5) when parasitized by *C. glomerata*, while they are in the late third (L3) instar for *C. rubecula*. This process eventually kills the caterpillar host (Brodeur et al. 1996).

### Experimental set-up

The insects used in this study were taken from lab-reared populations that were originally collected from agricultural fields in the surrounding of Wageningen University & Research, the Netherlands. Both *Pieris* species were reared and maintained on Brussels sprouts plants (*Brassica oleracea* L. var. *gemmifera*) in separate cages in a greenhouse compartment (21 ± 1°C, 25%–35% RH, 16:8 h light/dark). Male and female butterflies were allowed to freely mate in the cage and lay their eggs on the plants. Adult butterflies were fed with a saturated sugar solution. *Cotesia glomerata* and *C. rubecula* were reared in individual cages in distinct greenhouse compartments under the same conditions, utilizing *P. brassicae* caterpillars as hosts for both species. When the parasitoid larvae had pupated, pupae were collected and transferred to a smaller cage without plants. Emerged parasitoids were provided with 10% honey–water solution until they were used in the experiments.

When *P. brassicae* and *P. rapae* larvae had hatched, first instar larvae originating from the same egg-batch were collected from our rearing, separated in groups of similar size (*c*. 20 individuals) and subjected to three treatments: (1) parasitization by *C. glomerata*, (2) parasitization by *C. rubecula*, or (3) untreated (control caterpillars). Each caterpillar was individually parasitized as described in Cuny et al. (2022). In brief, caterpillars were considered parasitized when the parasitoid female had inserted its ovipositor in the caterpillars for at least 5 s for *C. glomerata* or 1 s for *C. rubecula*. Next, caterpillars from each combination of host and parasitoid species, as well as untreated caterpillars, were placed in separate cages on wild cabbage plants (*B. oleracea*, grown from seeds from Kimmeridge, UK; Gols et al. 2008) in the same greenhouse compartment (21 ± 1°C, 25%–35% RH, 16:8 h light/dark), until the caterpillars were used for microbiome sampling.

### Microbiome sampling

When parasitoid larvae were close to egression, eight caterpillars from each treatment were randomly picked from their respective cage for microbiome sampling (48 caterpillars in total; 8 × 2 caterpillar species × 3 treatments). At that time, caterpillars parasitized by *C. glomerata* were in the early fifth instar stage, while caterpillars parasitized by *C. rubecula* were in the late third instar stage. *Cotesia rubecula* is known to arrest host development at the third instar stage (Harvey et al. 1999), while *C. glomerata* allows its host to reach the final instar stage (Harvey et al. 2012). Nonparasitized *P. brassicae* and *P. rapae* caterpillars were in the early fifth instar stage. Preliminary analysis of a small number of *P. brassicae* caterpillars showed no significant variation in microbiome composition among the final instar stages. Caterpillars were collected using sterilized tweezers treated with 70% ethanol. Additionally, gloves were worn that were also sterilized with 70% ethanol before a caterpillar was sampled. Each caterpillar was put individually in a plastic sterile container (12 cm diameter; 5 cm height) containing tissue paper (to absorb frass and moisture) with a pierced lid. Caterpillars were starved overnight at room temperature in the same containers to allow the insects to empty their gut content. Subsequently, both the external (cuticle associated) and internal microbiome of the caterpillars and parasitoid larvae were sampled as described in Gloder et al. (2021) (Supplementary Table S1).

Briefly, the external microbiota of the caterpillars were collected by putting each caterpillar in a 2-ml microcentrifuge tube containing 1 ml of phosphate-buffered saline with 0.01% Tween80 (PBS-T), and vortexing it for 20 s. This washing solution was then used as a sample for the caterpillar’s external microbiome. Next, caterpillars were surface-sterilized with sodium hypochlorite (2.5%) and washed again two times in PBS-T (Gloder et al. 2021), and then dissected in the proximity of a Bunsen burner to obtain internal host and parasitoid larvae samples; Supplementary Fig. S1). Caterpillars were pinned onto a sterile dissection dish with flame-sterilized needles and cut open along the entire length of the caterpillar. Parasitoid larvae were collected with a sterilized pair of tweezers and put in a clean microcentrifuge tube. When necessary, some drops of sterile water were applied on top of the dissected caterpillars in order to ease the collection of the parasitoid larvae and to ensure that all larvae were retrieved; Supplementary Fig. S1). When caterpillars were parasitized by *C. glomerata* all the parasitoid larvae present in a single host were pooled and treated as a single sample. To avoid contamination of the parasitoid larvae with host microbes, the dissection was performed very carefully, aiming to not damage the host gut or any other tissues other than the host cuticle. Furthermore, dissection dishes were cleaned after each dissection, first with sodium hypochlorite (2.5%), then with ethanol (70%), and finally flooded with sterile water followed by air drying in sterile conditions. On average, 22.7 *C. glomerata* larvae (*c*. 2 mm in size) were recovered from *P. rapae* caterpillars (range: 8–37; median: 23), while 24.6 *C. glomerata* larvae were retrieved from *P. brassicae* caterpillars (range: 18–37; median 24). When caterpillars were parasitized by *C. rubecula*, in every host a single parasitoid larva was found (3–4 mm). The rest of the body of the caterpillars was then homogenized as described before (Gloder et al. 2021) to represent the internal host microbiome. Therefore, the remaining portion of each caterpillar was placed in a 2-ml tube containing a mixture of glass beads (three beads of 2 mm and two beads of 5 mm in diameter) and 1 ml PBS-T. The samples were then subjected to two consecutive cycles of 10 s at a speed of 5.5 m/s in a Bead Ruptor Elite (Omni international, Kennesaw, USA). The external and internal microbiome of the recovered parasitoid larvae were also sampled separately following the same protocol, but with a smaller working volume of PBS-T (500 µl instead of 1 ml).

### DNA extraction and molecular analysis

Genomic DNA was isolated from all external and internal samples (500 µl) using the PowerPro Soil Kit (Qiagen, Hilden, Germany) following the manufacturer’s instructions, with one modification: in the second step of the protocol the use of a vortex adapter was replaced by two cycles of 30 s (with a 10 s break in between) in the Bead Ruptor Elite at a speed of 5.5 m/s. Two negative controls in which the sample material was replaced by sterile, DNA-free water was included to confirm the absence of reagent contamination. DNA samples were then subjected to molecular analysis. First, bacterial presence and density was assessed by a qPCR (quantitative real-time Polymerase Chain Reaction) assay using the universal primers 515F and 806R (Caporaso et al. 2011), amplifying the V4 region of the bacterial 16S ribosomal RNA (rRNA) gene, as described previously (Gloder et al. 2021). Briefly, qPCR amplification was performed using the StepOnePlus™ RealTime PCR (Polymerase Chain Reaction) System (Applied Biosystems, Foster City, CA, USA). Each reaction mixture contained 0.2 µl of each primer (20 µM), 10 µl of the iTaq Universal SYBRGreen supermix (Biorad, Hercules, CA, USA), 8.6 µl of sterile distilled water, and 1 µl of template DNA. The thermal cycling protocol consisted of an initial denaturation step at 95°C for 2 min followed by 40 amplification cycles of 15 s at 95°C and 1 min at 60°C. Fluorescence (520 nm) was measured at the end of the elongation phase in each cycle. For each sample, the threshold cycle (C_T_) was calculated using StepOne™ software, and the baseline was set automatically above any noise. All qPCR reactions were performed in duplicate, and each run included a negative control where template DNA was replaced with sterile water. Additionally, a 10-fold dilution series of the targeted DNA fragment (ranging from 1 ng/µl to 1 fg/µl, measured with a Qubit fluorometer; Invitrogen, Carlsbad, USA) was included in each run to establish a calibration curve for calculating the number of gene copy numbers per µl DNA extract in the investigated samples (Lee et al. 2006). This dilution series was obtained by first amplifying the V4 region of a reference strain (*Pseudomonas* sp. ST09.08/02) using the primers 515F and 806R, and diluting it. The detection limit of the assay was set at a C_T_ value of 34, which corresponded to the lowest C_T_ value obtained for one of the blanks. Results of the gene copy numbers from the qPCR amplification are shown in Supplementary Table S2.

Additionally, for each sample the V4 region was amplified using Illumina barcoded versions of the same primers to assess the diversity and composition of the bacterial communities in the samples. Primers were designed according to Kozich et al. (2013) (dual index sequencing strategy) (Supplementary Table S3). In addition to the different DNA samples, three negative PCR controls (in which DNA template was replaced by DNA-free water) were included, as well as a DNA mock community sample that was composed of a number of bacterial species that likely occur in or on insects (Gloder et al. 2021) (Supplementary Table S4). PCR amplification, library preparation, sequencing, and bioinformatics analysis were performed as described previously (Gloder et al. 2021). Briefly, amplification was performed in a reaction volume of 40 µl, consisting of 2 µl DNA, 0.5 µM of each primer, 150 µM of each dNTP, 1 × Titanium Taq PCR buffer and 1 × Titanium Taq DNA polymerase (Takara Bio, Saint-Germain-en-Laye, France) with the following cycling protocol: 94°C for 120 s, followed by 35 cycles of 45 s at 95°C, 45 s at 59°C, and 45 s at 72°C, and a final elongation step of 10 min at 72°C. Amplicons from all insect samples and controls were purified using Agencourt AMPure XP magnetic beads (Beckman Coulter Genomics GmbH, South Plainfield, UK) following the manufacturer’s instructions. Subsequently, a Qubit high sensitivity fluorometer (Invitrogen) was used to measure the concentration of the purified amplicons, and each sample was pooled at equimolar concentrations. After ethanol precipitation, the amplicon library was loaded onto a 1.5% agarose gel, and the target band was excised and purified using a QIAquick Gel Extraction Kit (Qiagen). Following gel extraction, the concentration of the library was measured again, diluted to 2 nM, and then sent for sequencing at the Centre for Medical Genetics of the University of Antwerp (Antwerp, Belgium) using an Illumina MiSeq sequencer with a v2 500-cycle reagent kit (Illumina, San Diego, USA).

Bacterial sequences were received as demultiplexed FASTQ files, with barcodes and primer sequences removed. Paired-end reads were merged using USEARCH (v11.0.667) to generate consensus sequences (Edgar 2013), with no more than 10 mismatches allowed in the overlap region. Subsequently, reads shorter than 190 bp or with a total expected error threshold above 0.05 were discarded. Sequences were then classified into zero-radius operational taxonomic units (zOTUs; Edgar 2016), also known as amplicon sequence variants (Callahan et al. 2017) by the UNOISE3 algorithm as implemented in USEARCH (Edgar and Flyvbjerg 2015). The obtained dataset was decontaminated in R (v3.5.2) (R Core Team 2018) using microDecon (v1.0.2) (McKnight et al. 2019) to remove contaminants based on zOTU prevalence in the insect samples versus the mean of the three PCR controls (Davis et al. 2018). At the same time, the DNA extraction controls were removed from the dataset since they yielded only very low sequence numbers and no additional zOTUs in comparison with the PCR controls. Also, no band was obtained for the DNA extraction controls when loading the samples on an agarose gel, indicating that the DNA extraction kits were free of bacterial contamination. Subsequently, zOTUs occurring below a 0.1% relative abundance threshold in a given sample were discarded in that sample prior to further analysis (Gloder et al. 2021, Gorrens et al. 2022, Ijdema et al. 2022). In this way, analysis of the mock community only yielded the expected community members (Supplementary Table S5), demonstrating the robustness of our method. Finally, to correct for uneven sequence numbers, the number of sequences was rarefied to 2000 sequences per sample, while samples with less sequences were discarded from the analysis. The taxonomic origin of each zOTU was determined with the SINTAX algorithm as implemented in USEARCH based on the SILVA Living Tree Project v123. The identity of the most important zOTUs was also verified with a BLAST search in GenBank against type materials. When no significant similarity values were found (<97% identity), the BLAST analysis was performed against the entire database. Overall, results obtained by the BLAST analysis matched very well with those obtained with the SINTAX algorithm in USEARCH (Supplementary Table S5).

### Data analysis

Data analysis was performed on distinct datasets, one comprising samples from the caterpillars and another with samples from the parasitoid larvae. Additionally, the samples were categorized into internal and external samples. To test whether bacterial densities (determined by qPCR), expressed as the number of 16S rRNA gene copies per µl DNA extract, were affected by caterpillar host species (*P. brassicae* or *P. rapae*), parasitism status (parasitized by *C. glomerata*, parasitized by *C. rubecula*, or nonparasitized) and their interaction, a Scheirer–Ray–Hare test in rcompanion package in R (Mangiafico 2023) was performed for both the internal and external caterpillar samples (test performed on logarithmic values). This test is a nonparametric test used for a two-way factorial design (data did not meet the assumption of equal variances, as assessed with a Levene test). The same test was performed on samples collected from the parasitoid larvae residing within the parasitized caterpillars. For statistical analysis, samples in which bacteria could not be detected using qPCR but were detected through sequencing, were assigned to the qPCR detection threshold of 2.95 × 10^2^ 16S rRNA gene copies per µl DNA extract, which is equivalent to a C_T_ value of 34.

To assess whether the depth of our sequencing approach was sufficient to capture the bacterial diversity in the samples, rarefaction curves (Supplementary Fig. S2) were generated after rarefying the data to 2000 sequences per sample using the Phyloseq package in R (McMurdie and Holmes 2013, R Core Team 2018). The same package was used to determine zOTU richness (i.e. the number of observed zOTUs) and Shannon diversity for each sample. A two-way analysis of variance (ANOVA) was used to assess whether host caterpillar species, parasitism status, and their interaction affected zOTU richness and Shannon diversity in the caterpillar samples. The same analysis was performed to assess whether host caterpillar and parasitoid species, and their interaction, affected zOTU richness and Shannon diversity in samples from the parasitoid larvae. Bacterial community composition was visualized using nonmetric multidimensional scaling (NMDS) with the Bray–Curtis coefficient as distance measure in the R package vegan, based on relative abundance data. To test the hypothesis that caterpillar bacterial communities differed between host species and parasitism status, permutational analysis of variance (PERMANOVA) was performed on the same data set using the ‘adonis’ function in the vegan package (Oksanen et al. 2015). Host species, parasitism status, and their interaction were included as fixed factors in the analysis. Similarly, PERMANOVA was performed on the parasitoid larvae data to assess whether bacterial community composition within and on the parasitoid larvae differed between host caterpillars and parasitoid species, and whether there was an interaction effect. Statistical significance was tested using 1000 permutations. This analysis and the NMDS visualization were repeated on a reduced dataset where zOTUs belonging to the same family were merged into family-level phylotypes. The sequence data obtained in this study has been submitted in the Sequence Read Archive at NCBI under Bioproject PRJNA1082293.

Indicator species analyses using the R package ‘indicspecies’ were performed to investigate whether zOTUs could be assigned to specific treatments. Analyses were performed separately for each caterpillar species and for external and internal microbiomes. A complementary co-occurrence matrix was calculated and visualized using the ‘co-occur’ R package (Griffith et al. 2016) using the same datasets. Finally, Kruskal–Wallis tests were used to assess whether the relative abundance of individual zOTUs differed significantly among treatments. Analyses were restricted to the 21 most abundant zOTUs, occurring at a mean relative abundance >1% in at least one of the caterpillar treatment groups.

## Results

### Bacterial density

**Figure 1.**
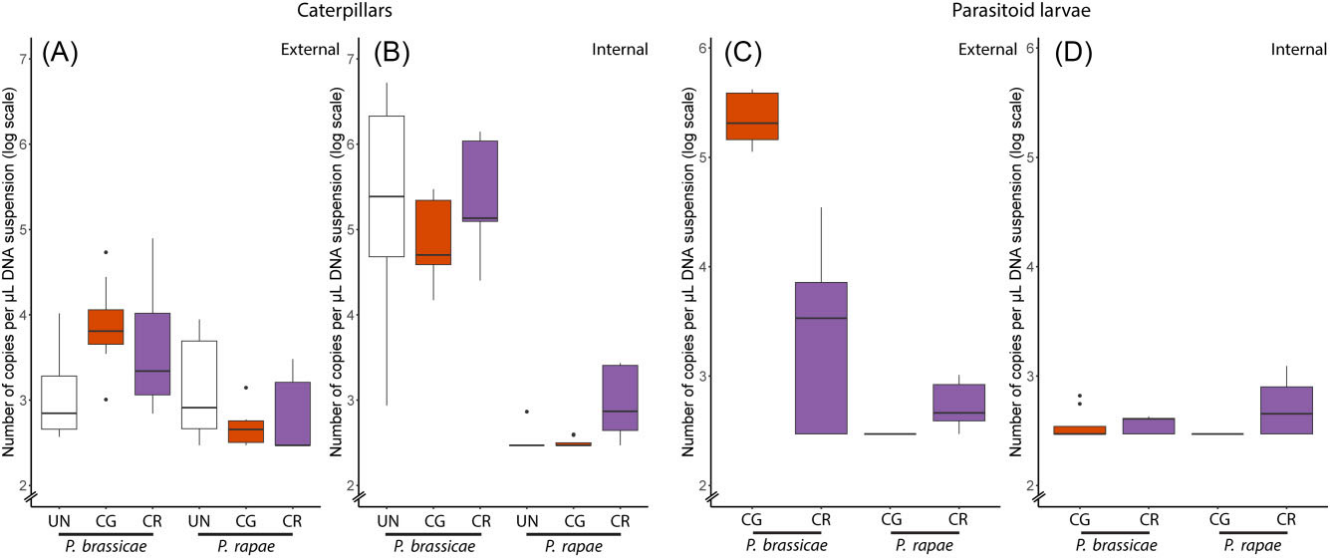
Boxplots showing the numbers of bacterial 16S rRNA gene copies per µl DNA suspension (logarithmic scale) in the external (A) and internal (B) microbiomes of the investigated caterpillars, as well as in the external (C) and internal (D) microbiomes of the parasitoid larvae collected. *Pieris brassicae* and *P. rapae* caterpillars were parasitized with either *C. glomerata* (CG) or *C. rubecula* (CR), or remained unparasitized (UN). Samples that were below the detection limit were assigned 2.95 × 10^2^ 16S rRNA gene copies per µl DNA extract, which corresponds to the qPCR detection threshold. The lower and upper whiskers correspond to the minimum and maximum values, with the bar in the middle marking the median value while dots represent outliers.

Absolute bacterial densities (calculated by qPCR) were significantly higher for caterpillars of *P. brassicae* than for those of *P. rapae*, both externally and internally, and parasitism status did not impact this result (Table 1; Fig. 1A and B). Larvae from both parasitoid species had a higher external bacterial density when infecting *P. brassicae* than when infecting *P. rapae*. This difference was more pronounced in *C. glomerata* larvae than in *C. rubecula* larvae (Table 1; Fig. 1C). For the internal samples of the parasitoid larvae, regardless of host species, there was a slightly, but significantly higher internal bacterial density in larvae from *C. rubecula* than in larvae from *C. glomerata* (Table 1; Fig. 1D).

**Table 1.** Results of Scheirer Ray Hare analysis on bacterial densities in the external (ext) and internal (int) samples of the investigated caterpillars and parasitoid larvae. Significant differences (*P* < .05) are indicated in bold.

	Caterpillars				Parasitoid larvae			
	Ext (*n* = 43)		Int (*n* = 46)		Ext (*n* = 43)		Int (*n* = 46)	
	*H*	*p*	*H*	*p*	*H*	*p*	*H*	*p*
Host	10.231	**.001**	33.520	**<.001**	13.290	**<.001**	0.505	.477
Parasitism status (caterpillars)/Parasitoid species (parasitoid larvae)	0.769	.680	1.477	.478	0.895	.344	4.424	**.035**
Host: parasitism status (caterpillars)/Host: parasitoid species (parasitoid larvae)	5.841	.054	0.564	.754	8.845	**.003**	0.833	.361

### Bacterial diversity and community composition

**Figure 2.**
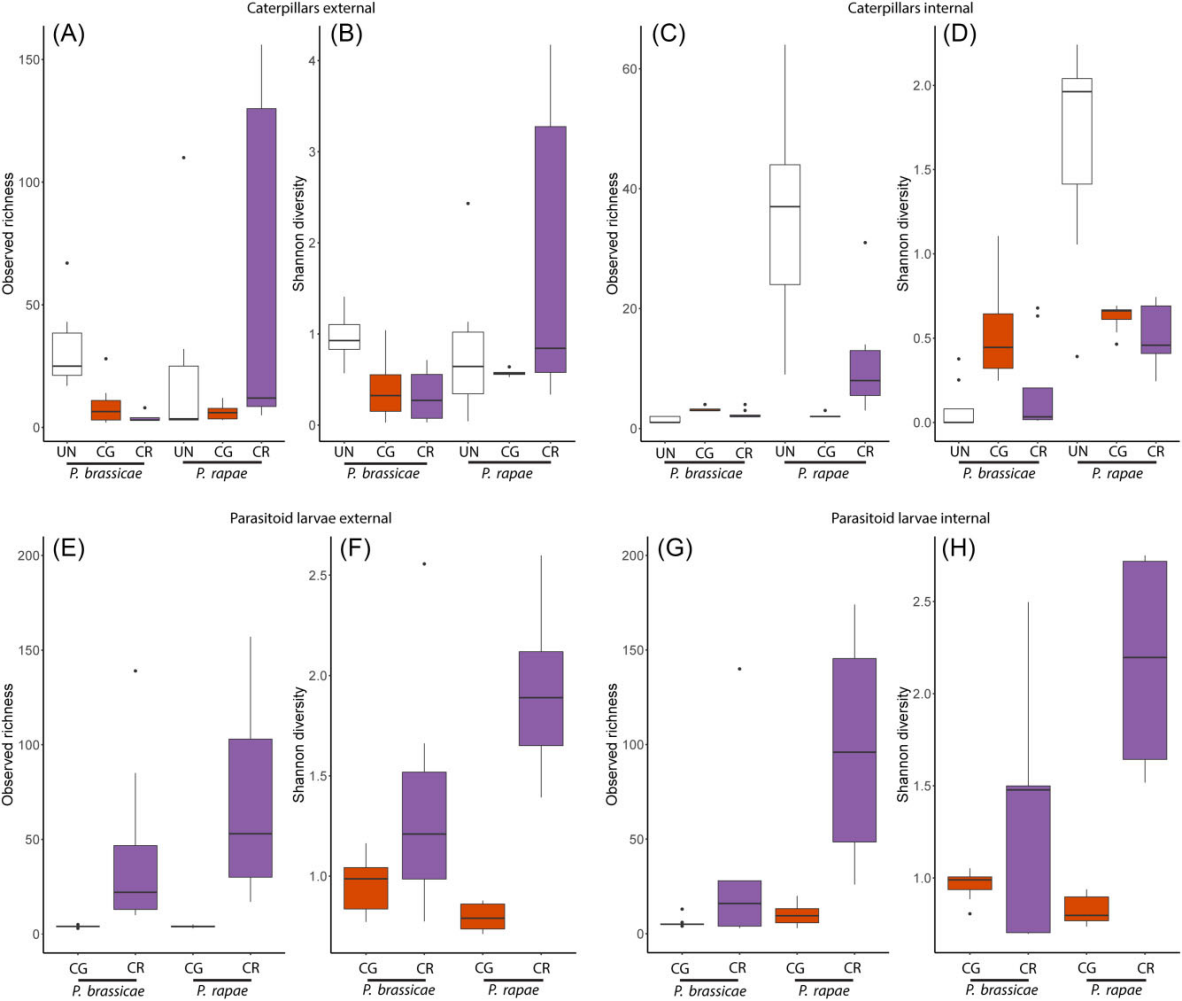
Boxplots showing alpha diversity (zOTU richness and Shannon index) comparisons of the external and internal microbiomes of the different (A–D) caterpillars and (E–H) parasitoid larvae samples studied. *Pieris brassicae* and *P. rapae* caterpillars were parasitized with either *C. glomerata* (CG) or *C. rubecula* (CR), or remained unparasitized (UN). The lower and upper whiskers correspond to the minimum and maximum values, with the bar in the middle marking the median value while dots represent outliers.

After quality filtering, removal of potential contaminants and rarefying to 2000 sequences per sample, a total of 658 zOTUs were retained in the analysis (Supplementary Table S5), covering a total of 144 samples (Supplementary Table S1). In general, rarefaction curves approached saturation (Supplementary Fig. S2), indicating that our sequencing depth of 2000 reads per sample was sufficient to cover the bacterial diversity in the samples. Two-way ANOVA of the caterpillar external microbiomes revealed no significant differences in zOTU richness between the two host caterpillars (Fig. 2A), while a significant difference was found in Shannon diversity (Fig. 2B; Table 2). This indicates that while the number of bacterial species is similar, the distribution and abundance of those species differ between the caterpillar hosts.

**Table 2.** Results of two way ANOVA on the observed bacterial zOTU richness and Shannon diversity in the investigated caterpillars and parasitoid larvae. Significant differences (*P* < .05) are indicated in bold.

	Caterpillars							
	External (*n* = 43)				Internal (*n* = 43)			
	Richness		Shannon		Richness		Shannon	
	*F*	*p*	*F*	*p*	*F*	*p*	*F*	*p*
Host	3.390	.074	5.887	**.020**	29.270	**<.001**	45.547	**<.001**
Parasitism status	2.143	.131	2.102	.136	13.160	**<.001**	7.708	**.001**
Host: parasitism status	4.562	**.017**	4.803	**.014**	18.020	**<.001**	21.911	**<.001**
	**Parasitoid larvae**							
	**External (*n* = 30)**				**Internal (*n* = 25)**			
	**Richness**		**Shannon**		**Richness**		**Shannon**	
	***F***	***p***	***F***	***p***	***F***	***p***	***F***	***p***
Host	0.557	.462	0.846	.366	2.099	.162	0.840	.369
Parasitoid species	16.800	**<.001**	30.918	**<.001**	14.571	**.001**	24.074	**<.001**
Host: parasitoid species	1.256	.272	7.619	**.010**	3.325	.082	7.218	**.014**

Although parasitism did not significantly affect zOTU richness or Shannon diversity in the external caterpillar samples, *P. rapae* caterpillars parasitized with *C. rubecula* showed a higher bacterial richness and diversity (Table 2; Fig. 2A and B). The internal microbiomes showed significant differences between the two caterpillar species, both in terms of zOTU richness and Shannon diversity. Higher numbers of bacterial zOTUs and greater diversity were found in *P. rapae* than in *P. brassicae* (Table 2; Fig. 2C and D). Furthermore, parasitism had a significant effect on the internal caterpillar microbiomes, with a more pronounced effect in *P. rapae* than in *P. brassicae*, both for richness and diversity. Nonparasitized *P. brassicae* contained an average of 1.4 (range 1–2) zOTUs, which increased to 3.3 (range 3–4) when parasitized by *C. glomerata* and to 2.4 (range 2–4) when parasitized by *C. rubecula*. In contrast, uninfected *P. rapae* caterpillars harboured an average of 35.1 (range 9–64) zOTUs, while this was only 2.1 (range 2–3) and 11.3 (3–46) when parasitized with *C. glomerata* and *C. rubecula*, respectively (Table 2; Fig. 2C and D). Parasitoid larvae had a higher zOTU richness and Shannon diversity in both the external and internal samples of *C. rubecula* compared to *C. glomerata*, and this difference in diversity was more pronounced when parasitizing *P. rapae* than when parasitizing *P. brassicae* (Table 2; Fig. 2E–H).

**Figure 3.**
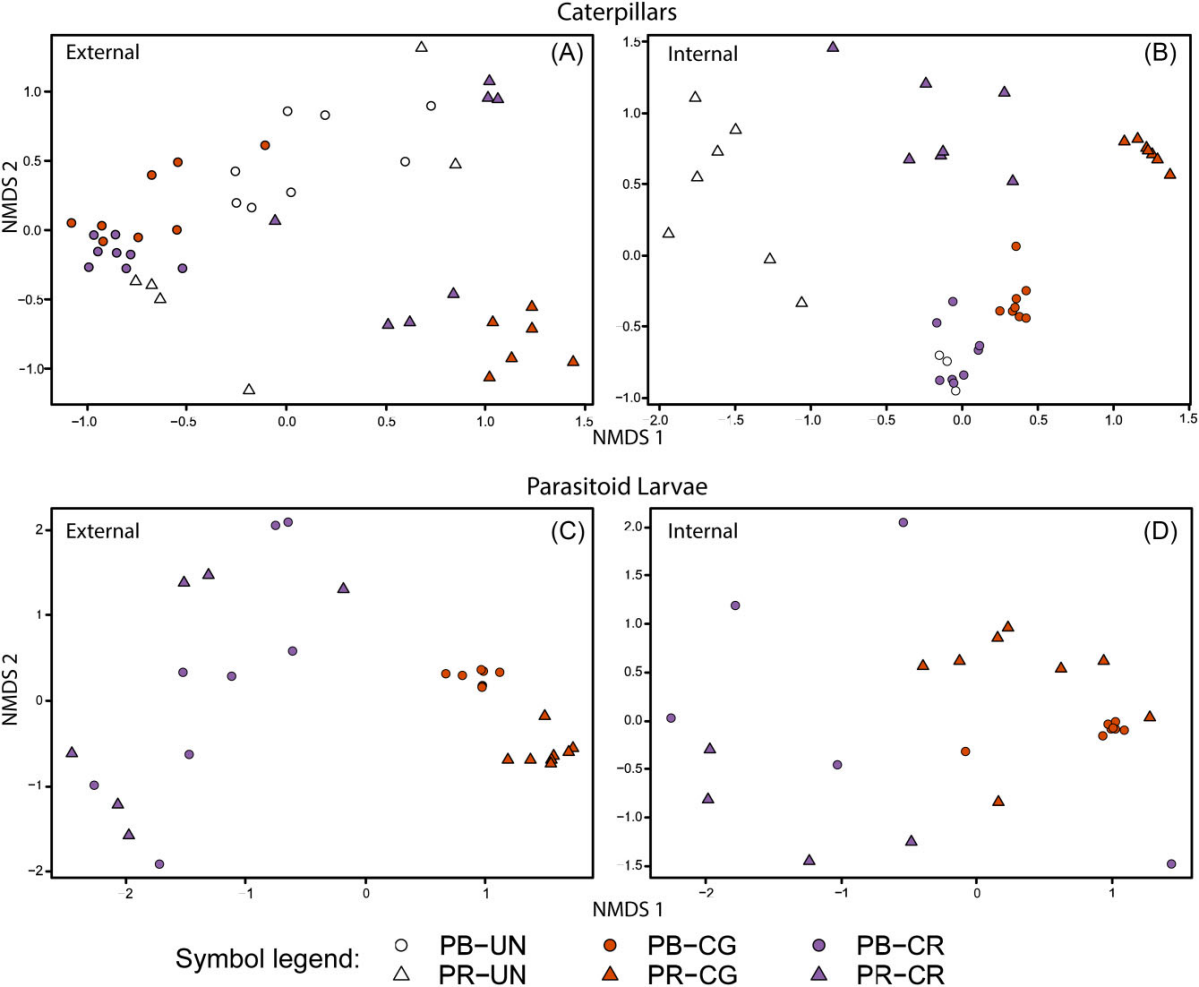
NMDS ordination plots based on Bray–Curtis distances of relative abundance data of the external and internal microbiomes of the different caterpillars (A and B) and parasitoid larvae (C and D) samples studied. *Pieris brassicae* (PB) (circles) and *P. rapae* (PR) (triangles) caterpillars were parasitized with either *C. glomerata* (CG) or *C. rubecula* (CR), or remained unparasitized (UN). Stress values of the plots are 0.165 (A), 0.109 (B), 0.117 (C), and 0.154 (D).

PERMANOVA analyses (Table 3; Supplementary Table S6) showed significant differences in both the external and internal bacterial community composition between caterpillars of *P. brassicae* and *P. rapae*, as well as between the different treatments (Table 3; Fig. 3A and B). However, the effect of parasitism was more pronounced in samples from *P. rapae* compared to *P. brassicae* (Table 3; Fig. 3A and B). The external microbiome of parasitoid larvae also differed significantly between both parasitoid species and between larvae collected from *P. brassicae* and *P. rapae* (Table 3; Fig. 3C). Moreover, the interaction between host species and parasitoid species was statistically significant for the external parasitoid samples. In contrast, there was a significant difference between the internal microbiome of larvae of the two parasitoid species (Table 3; Fig. 3D), while no significant differences were found between host species, nor was there a significant interaction effect (Table 3; Supplementary Table S6). When repeating the analysis at the family level, the same patterns were observed (Supplementary Fig. S3; Supplementary Table S7).

**Table 3.** Results of PERMANOVA on the external and internal bacterial community composition of the investigated caterpillars and parasitoid larvae. Significant differences (*P* < .05) are indicated in bold.

	Caterpillars			
	External (*n* = 43)		Internal (*n* = 46)	
	*F*	*p*	*F*	*p*
Host	51.795	**<.001**	77.020	**<.001**
Parasitism status	8.121	**<.001**	15.606	**<.001**
Host: parasitism status	9.618	**<.001**	8.452	**<.001**
	**Parasitoid larvae**			
	**External (*n* = 30)**		**Internal (*n* = 25)**	
	***F***	***p***	***F***	***p***
Host	29.037	**<.001**	0.824	.503
Parasitoid species	21.320	**<.001**	25.063	**<.001**
Host: parasitoid species	7.057	**<.001**	2.507	.056

### Taxonomic classification, incidence, and relative abundance of caterpillar–host microbes

**Figure 4.**
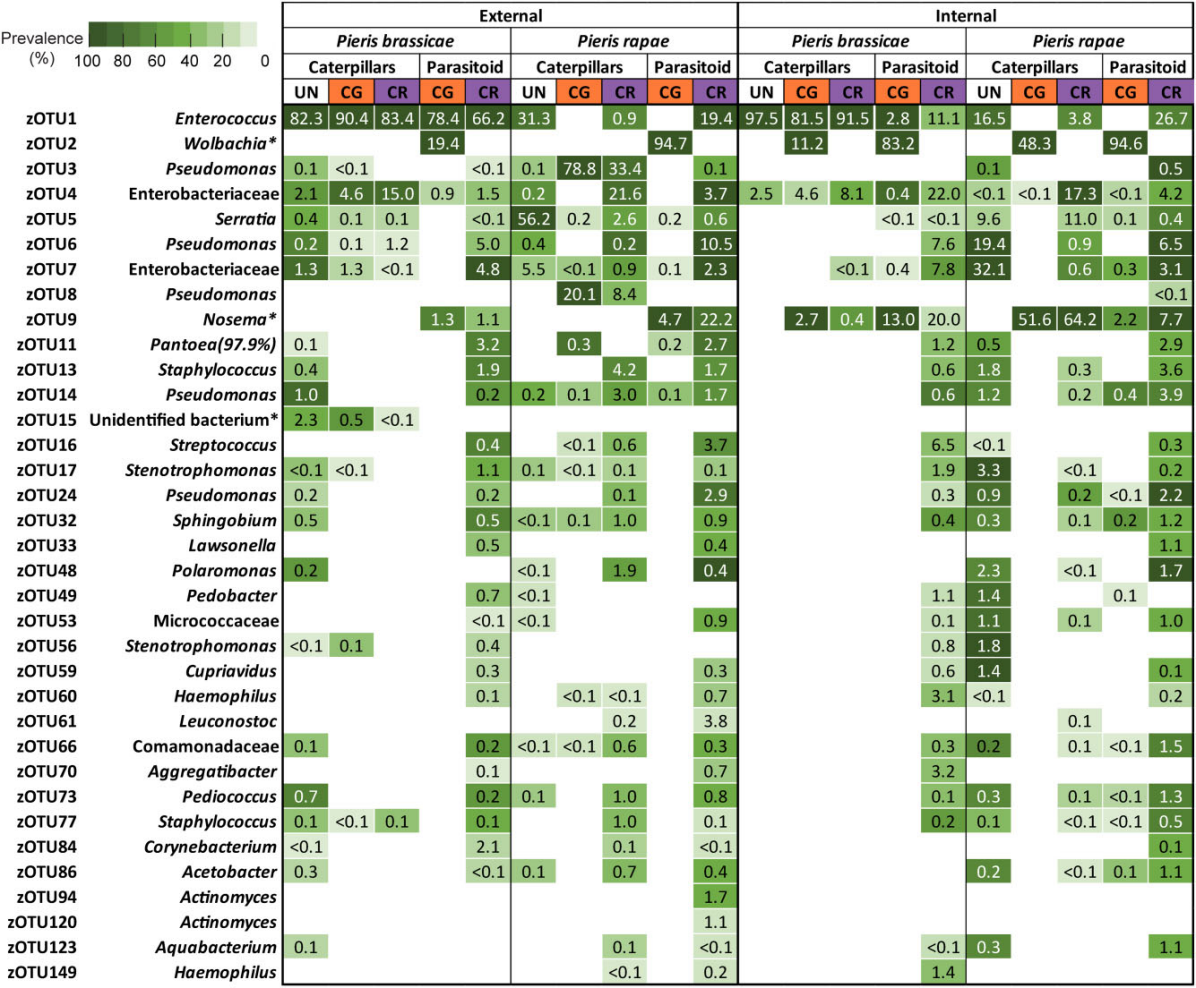
Bacterial community profiles of the investigated caterpillars and parasitoid larvae. *Pieris brassicae* and *P. rapae* caterpillars were parasitized with either *C. glomerata* (CG) or *C. rubecula* (CR), or remained unparasitized (UN). Bacterial taxa represent the most prevalent taxa in the different subgroups based on host caterpillar and parasitism status for caterpillars and host caterpillar and parasitoid species for parasitoid larvae (present at a mean relative abundance >1% in at least one subgroup). For each zOTU, the average relative abundance for each subgroup is given in the box as a percentage, whereas the colour indicates prevalence (white is absent). zOTUs are identified by a BLAST search against type materials in GenBank. When no significant similarity was found with type materials, the BLAST analysis was performed against entire GenBank (indicated with and asterisk). Identifications were performed at genus level; when identical scores were obtained for different genera, identifications were performed at family level. When identity percentages were lower than 99%, the percentage of sequence identity with the GenBank entry is given between brackets. Hits with uncultured bacteria are indicated as unidentified bacterium.

Bacteria found on and inside the analysed caterpillars represented several environmental and insect-associated species belonging to diverse phyla, with the most abundant species belonging to *Pseudomonadota* (Proteobacteria), *Bacillota* (Firmicutes), and *Actinomycetota* (Actinobacteria) (Supplementary Table S5). In general, caterpillar microbiomes were dominated by a limited number of bacterial species (Fig. 4; Supplementary Fig. S4). In particular, irrespective of parasitism status, both the external and internal microbiomes of *P. brassicae* caterpillars were dominated by a single zOTU (zOTU1), identified as *Enterococcus* sp. This bacterium was found at an average relative abundance of 84.1% and 90.2% on and inside *P. brassicae* caterpillars, respectively, while it was less abundant on (10.2%) and inside (6.4%) *P. rapae* caterpillars. Moreover, the bacterium was present in all analysed *P. brassicae* samples, but was absent in any *P. rapae* caterpillar sample parasitized by *C. glomerata* (Fig. 4). In the external microbiome, *Enterococcus* sp. was found in five out of six nonparasitized *P. rapae* caterpillars and in five out of seven caterpillars parasitized by *C. rubecula*, while in the internal microbiome it was found in six out of seven nonparasitized individuals and in five out of seven individuals parasitized by *C. rubecula* (Fig. 4).

A few bacterial species were common and abundant on or inside nonparasitized *P. rapae* caterpillars, while they were rare or absent on or inside *P. rapae* caterpillars that were parasitized. In the external microbiome, zOTU5, identified as *Serratia* sp., was consistently present on all nonparasitized *P. rapae* caterpillars with an average relative abundance of 56.2%, while it was detected on only a few parasitized individuals at a lower relative abundance. Additionally, zOTU7, an unidentified member of the Enterobacteriaceae family, and zOTU6, identified as *Pseudomonas* sp., were both present in all internal samples from nonparasitized *P. rapae* caterpillars, where they occurred at an average relative abundance of 32.1% and 19.4%, respectively. In contrast, they were not or only sporadically detected in parasitized individuals (Fig. 4). Conversely, the external microbiome of parasitized *P. rapae* caterpillars showed some bacterial species that were more frequently present than others. Specifically, zOTU3 and zOTU8, both belonging to the genus *Pseudomonas*, were abundantly present on parasitized individuals, while they were only found at low relative abundances in nonparasitized caterpillars (<0.1%) (Fig. 4). One bacterial zOTU (zOTU2) was exclusively present in the internal microbiome of *C. glomerata*-parasitized caterpillars and absent in any other sample. Moreover, it was found in every *C. glomerata*-parasitized individual analysed (Fig. 4). This bacterium, identified as *Wolbachia pipientis*, occurred at an average relative abundance of 11.2% in *C. glomerata*-parasitized *P. brassicae* caterpillars and 48.3% in *P. rapae* caterpillars parasitized with *C. glomerata*. Further, zOTU9, identified as *Nosema* sp., a microsporidium that possesses a ribosomal unit similar to bacteria (Kawakami et al. 1992), was frequently found in parasitized caterpillars. In particular, it was present in all internal samples of *C. glomerata*-parasitized caterpillars with a relative abundance of 2.7% and 51.6% in *P. brassicae* and in *P. rapae* hosts, respectively. This zOTU was also found in five of the eight investigated *C. rubecula*-parasitized *P. brassicae* individuals (with an average relative abundance of 0.4%) and in all *C. rubecula*-parasitized *P. rapae* individuals (with an average relative abundance of 64.2%). In contrast, this *Nosema* species was not detected in any of the nonparasitized caterpillars or in any external samples of the parasitized caterpillars (Fig. 4).

Indicator species analysis confirmed that some bacterial species were specific to some treatment groups. In particular, for the internal microbiome, *Wolbachia* (zOTU2) and *Nosema* (zOTU9) were identified as indicators of *C. glomerata*-parasitized caterpillars of both host species. *Nosema* (zOTU9) was also highlighted as an indicator of *C. rubecula*-parasitized *P. rapae* caterpillars (Supplementary Table S8). Co-occurrence analysis of the external microbiome of *P. brassicae* caterpillars showed that zOTU4 (Enterobacteriaceae) negatively correlated with ten other zOTUs, suggesting that its presence interferes with the growth of other bacteria. Similarly, in the external microbiome of *P. rapae*, the *Pseudomonas* species corresponding to zOTU8 was negatively correlated with six other species. In the internal microbiome, a strong positive co-occurrence was observed between *Wolbachia* (zOTU2) and *Nosema* (zOTU9) in both host species. In contrast, a negative co-occurrence was found between these two species and several zOTUs in *P. rapae* (Supplementary Fig. S5). Kruskal–Wallis analyses performed on single zOTUs confirmed significant differences in relative abundances between treatments for several zOTUs, especially in the internal microbiome of both host species where abundances of zOTU2 (*Wolbachia*) and zOTU9 (*Nosema*) were significantly higher in parasitized than in nonparasitized individuals (Supplementary Table S9).

### Taxonomic classification, incidence, and relative abundance of parasitoid-larvae microbes

The same bacteria found abundantly in the internal compartments of parasitized hosts also dominated the microbiomes of parasitoid larvae (Fig. 4; Supplementary Fig. S4). Particularly, the external microbiome of *C. glomerata* larvae collected from *P. brassicae* caterpillars was dominated by both *Enterococcus* (zOTU1) (incidence of 100%; average relative abundance of 78.4%) and *Wolbachia* (zOTU2) (100%; 19.4%). When collected from *P. rapae*, the external microbiome of *C. glomerata* was particularly dominated by the *Wolbachia* zOTU, with an average relative abundance of 94.7% (Fig. 4). The internal microbiome of *C. glomerata* larvae was mainly dominated by *Wolbachia*, irrespective of the host caterpillar, with a relative abundance of 83.2% in *P. brassicae* and 94.6% in *P. rapae*. Additionally, *C. glomerata* larvae in *P. brassicae* contained a substantial fraction (13.0%) of *Nosema* (zOTU9), which was also present in larvae from *P. rapae*, but at a lower average relative abundance (2.2%) (Fig. 4).

Similarly, in *C. rubecula* larvae, a few zOTUs dominated the microbial communities. In the external microbiome of *C. rubecula* larvae collected from *P. brassicae, Enterococcus* (zOTU1) was the most abundant bacterium, with an average relative abundance of 66.2%. For individuals collected from *P. rapae*, this *Enterococcus* zOTU had a relative abundance of 19.4%, and *Nosema* (zOTU9) and *Pseudomonas* sp. (zOTU6) were also abundantly present (Fig. 4). The internal microbiome of *C. rubecula* larvae mainly contained *Nosema* (zOTU9) and *Enterococcus* sp. (zOTU1), irrespective of their host, along with several other bacteria that occurred at lower relative abundances. In larvae collected from *P. brassicae*, these zOTUs had a mean relative abundance of 20.0% and 11.1%, respectively. When *P. rapae* was the host, the relative abundances were 7.7% for *Nosema* and 26.7% for *Enterococcus* (Fig. 4).

## Discussion

### Bacteria are commonly present in and on host caterpillars and developing parasitoid larvae

Although the effects of parasitism on host microbial communities have been increasingly studied in recent years, particularly in lepidopteran hosts (Cavicchiolli de Oliveira and Consoli 2020, Gloder et al. 2021, Zhang et al. 2022), little is still known about how host microbial communities and those of developing parasitoid larvae are influenced by both their host and the parasitoid species. Here, through estimation of bacterial abundance by qPCR, we found that bacteria were commonly present in and on the investigated caterpillars, especially in *P. brassicae*, confirming our previous findings (Gloder et al. 2021). Furthermore, high-throughput sequencing of 16S rRNA genes revealed that the bacterial microbiome of *P. brassicae* and *P. rapae* caterpillars was mainly composed of Pseudomonadota (Proteobacteria), Bacillota (Firmicutes), and Actinomycetota (Actinobacteria), which are the most common phyla found in lepidopteran species, including *Pieris* spp. (Robinson et al. 2010, Gao et al. 2019, Wang et al. 2020, Gloder et al. 2021).

Overall, caterpillar microbiomes were strongly dominated by an *Enterococcus* species (zOTU1), with an average relative abundance of up to 97.5% in *P. brassicae* caterpillars. Although our rarefaction curves tended to reach saturation, presumably a greater sampling depth might still be required to cover the full diversity of the microbiome in these samples. The strong dominance of this *Enterococcus* zOTU may have led to under-amplification of other bacterial DNA (Mayerhofer et al. 2020). Although this bacterium was not found in field-collected *P. brassicae* caterpillars (Gloder et al. 2021), this result is consistent with a previous study, where the same *Enterococcus* zOTU was strongly associated with lab-reared *P. brassicae* caterpillars (Bourne et al. 2023). The high relative abundance of this species in lab-reared caterpillars may be linked to the controlled laboratory conditions under which the caterpillars were reared and maintained, which were the same in both studies. Our results also show that the parasitoid larvae collected from the caterpillars possess their own microbiota. However, results also showed that the external microbiome of the parasitoid larvae shares some similarities with the internal microbiome of the caterpillar host species, suggesting that there may be an interaction and exchange between the two microbiomes.

### Parasitism alters the microbial community composition of host caterpillars: crucial role of host identity

Our results clearly show that parasitism by *Cotesia* parasitoids significantly alters both the internal and the external microbial community composition of host caterpillars, and that these effects are strongly dependent on the host. In a previous study (Gloder et al. 2021), parasitism of *P. brassicae* by *C. glomerata* altered the internal microbiome of the caterpillars, but no effects were observed on the external microbiome, possibly because that study focused on field-collected insects. Differences in microbiomes between natural and lab-reared insect populations have been observed frequently, and are most probably due to factors like diet and environmental conditions (Park et al 2019, Wang et al. 2019, Martínez-Solís et al. 2020). We found a strong host-dependent variation in the occurrence of the *Enterococcus* zOTU (zOTU1). While it remained at high relative abundance on and in parasitized *P. brassicae* caterpillars, its relative abundance was drastically lowered on and in parasitized *P. rapae* caterpillars compared to nonparasitized caterpillars, irrespective of the parasitoid species. Instead, a higher relative abundance of species belonging to the *Pseudomonas* genus (zOTU3 and zOTU8) was detected in the external microbiome of parasitized *P. rapae* individuals, along with a diminished presence of a *Serratia* species (zOTU5) that was highly abundant on nonparasitized individuals. Some *Pseudomonas* and *Serratia* species are known as beneficial bacteria (Teoh et al. 2021, Pons et al. 2022), while others may be insect pathogens (Pineda-Castellanos et al. 2015, Flury et al. 2016). It is unclear what effect these bacteria had on their host in this study. It has been suggested that *C. rubecula* is better adapted to *P. rapae* than to *P. brassicae* due to differences in host physiology and/or the ability of the parasitoid to regulate these (Harvey et al. 1999). Variation in host physiology between *P. brassicae* and *P. rapae* may also have favoured specific microbes in one host, while adversely affecting them in the other. Further research is needed to investigate this.

### Parasitism alters the microbial community composition of host caterpillars: crucial role of parasitoid identity

In addition to host-dependent variation, our results show that parasitism-induced changes in the host microbiome are also determined by the parasitoid species. This is particularly clear for the internal microbiome of caterpillars parasitized by *C. glomerata*. Specifically, we found that both *P. brassicae* and *P. rapae* caterpillars parasitized with *C. glomerata* contained a substantial fraction of *Wolbachia* (zOTU2), which was not detected in nonparasitized caterpillars or in caterpillars parasitized with *C. rubecula*. Furthermore, our co-occurrence analysis indicated that this zOTU was negatively associated with several zOTUs in *P. rapae* parasitized caterpillars. The relative abundance of *Wolbachia* was also higher in parasitized *P. rapae* caterpillars (48.3%) compared to parasitized *P. brassicae* caterpillars (11.2%). However, when comparing the absolute abundance of *Wolbachia*, estimated by multiplying its relative abundance by the 16S rRNA gene copy number per ul of DNA in each sample, *P. brassicae* had 1.31 × 10^4^ gene copies of *Wolbachia* per µl of DNA sample, whereas *P. rapae* had 1.54 × 10^2^ gene copies per µl of DNA extract. This suggests that even though the relative abundance of *Wolbachia* was low in *P. brassicae*, the bacterium still had a high concentration, higher than in *P. rapae*, which had a lower overall bacterial density. In addition, *Wolbachia* was abundantly found in the developing *C. glomerata* larvae inside the caterpillar hosts, reaching an average relative abundance of 83.2% and 94.6% in parasitoid larvae in *P. brassicae* and *P. rapae* hosts, respectively.

*Wolbachia* is a well-studied genus of intracellular endosymbionts that are commonly found in arthropods. These bacteria often manipulate host reproduction to favour their own transmission (Werren et al. 2008, Sanaei et al. 2020) and can benefit their hosts by providing resistance against insecticides and viruses (Berticat et al. 2002, Hedges et al. 2008). *Wolbachia* is estimated to be present in about 80% of lepidopteran species, including species belonging to the Pieridae family (Ahmed et al. 2015a). However, in our study, *Wolbachia* was not detected in nonparasitized individuals of *P. rapae* or *P. brassicae*, nor in nonparasitized *P. brassicae* individuals in previous studies (Gloder et al. 2021, Bourne et al. 2023). PCR analysis using *Wolbachia*-specific primers (Doudomis et al. 2012) revealed the presence of this bacterium in adult females of our *C. glomerata* rearing but not in females of *C. rubecula*, confirming previous results (Rattan et al. 2011, Dicke et al. 2020, Gloder et al. 2021). Therefore, it is reasonable to assume that *C. glomerata* transferred *Wolbachia* into the caterpillars during oviposition after which it established and replicated, explaining its high relative abundance in parasitized caterpillars. This is in line with previous studies showing that parasitoids may transfer *Wolbachia* into their host during oviposition (Ahmed et al. 2015b). Alternatively, *Wolbachia* may be derived from the parasitoid eggs or developing larvae within the host caterpillars, allowing the parasitoid to pass essential symbionts to the next generation, although little is known to support this hypothesis. The presence of *Wolbachia* in adult parasitoids could benefit the wasps by enhancing host-searching ability and oviposition frequency (Furihata et al. 2015). However, *Wolbachia* may also have negative effects on parasitoids by increasing their susceptibility to hyperparasitoids, i.e. parasitic wasps that attack the larvae and pupae of primary parasitoids (van Nouhuys et al. 2016). Recent research has suggested that the presence of *Wolbachia* in parasitized caterpillars changes their body odours, providing reliable cues for hyperparasitoids to locate potential hosts (Bourne et al. 2023). Likewise, conspecifics of the primary parasitoid may use these signals to avoid parasitized hosts (Cusumano et al. 2020), but further research is needed to confirm this. While *Wolbachia* was exclusively associated with caterpillars parasitized with *C. glomerata*, a *Nosema* species (zOTU9) was abundantly present within parasitized caterpillars, irrespective of the host or parasitoid species. The species was also abundantly present in developing parasitoid larvae, while it was not found in nonparasitized caterpillars or the external microbiome of the parasitized caterpillars. Additional PCR analysis using *Nosema* specific primers (Bosmans et al. 2018) on adult females of *C. glomerata* and *C. rubecula* from our rearing showed that the *Nosema* zOTU was also present in several analysed wasps (Supplementary Fig. S6), suggesting that *Nosema* was transferred from the parasitoids to the caterpillars during oviposition. This *Nosema* zOTU was probably introduced in our rearing by renewing the parasitoid cultures with field-collected individuals. Unlike *Wolbachia, Nosema* is an intracellular microsporidian parasite, recently reclassified as a fungus, that is capable of infecting a wide range of insects (Yaman et al. 2014, Ia et al. 2017, Bosmans et al. 2018, Galajda et al. 2021). Although being an eukaryote, *Nosema* has a number of prokaryotic features, particularly in its ribosomes (Kawakami et al. 1992). A BLAST analysis against GenBank revealed that the two primers used in this study perfectly matched with the small subunit rRNA gene of *Nosema*, explaining its presence in our data set. The sequence obtained in our study showed a 100% match with *Nosema pieriae*, a common pathogen in *Pieris* butterflies (Choi et al. 2002, Yaman et al. 2014). The proliferation of this opportunistic pathogen could have been favoured in parasitized individuals as it is known that parasitism causes reduced host immunity responses, which may also affect microbial growth (Cavichiolli de Oliveira and Consoli 2020). Additionally, the presence of this microbial parasite might have benefitted the development of the parasitoids by weakening their host (Mabbott 2018), although further research is needed to confirm this scenario.

Although the exact mechanisms driving parasitoid-dependent alterations in host microbiomes remain unclear, our data strongly suggest that parasitoid-associated microorganisms can be transferred from the parasitoid to the caterpillars during oviposition or originate from the developing parasitoid larvae. Many parasitoids release effectors (i.e. molecules that facilitate successful parasitism) into the host that impair the immune system of their hosts. Maternally transmitted effectors, such as symbiotic viruses and venom, are injected during oviposition (Dicke et al. 2020). Other effectors, not transmitted by the female parasitoid, include teratocytes (i.e. autonomous cells that detach from the egg membrane during hatching; Strand 2014) and secretions released by the parasitoid larvae (Pang et al. 2023). These effectors could, in turn, influence the host microbiome by modulating the host immune system and physiology. Further research is needed to find out how important they are in shaping the microbiome of host insects.

## Conclusions

In summary, our findings demonstrate that endoparasitism by koinobiont parastoids significantly affects both the internal and external microbial communities of host caterpillars, and that such changes depend both on the host and parasitoid species. Our results also show that the developing *C. glomerata* and *C. rubecula* larvae have distinct microbial communities. The internal microbiome of *P. brassicae* and *P. rapae* caterpillars parasitized by *C. glomerata* consistently harboured *Wolbachia*, which was entirely absent in nonparasitized individuals or those parasitized by *C. rubecula*. Additionally, parasitized caterpillars showed a high relative abundance of *Nosema pieriae*, particularly in *P. rapae* caterpillars. Further investigations are warranted to unravel the potential roles of these microbes in the intricate interactions among the host caterpillar, the parasitoid, and higher trophic levels.

## Supplementary Material

fiae115_Supplemental_File
